# MDP-salts as an adhesion promoter with MDP-primers and self-adhesive resin cement for zirconia cementation

**DOI:** 10.1186/s12903-023-03663-y

**Published:** 2023-11-22

**Authors:** Ahmed Abdou, Nasser Hussein, Nour E. A. Abd El-Sattar, Tomohiro Takagaki, Citra Kusumasari, Amr Rizk, Emad A. Abo-Alazm

**Affiliations:** 1 Faculty of Dentistry, Al-Ayen University, Thi-Qar, Nasiriyah, Iraq; 2 Fixed Prosthodontics Department, Faculty of Dentistry, Modern University for Technology, and Information, Mokatam, Cairo, Egypt; 3 Department of Chemistry, Organic Labs, Faculty of Science, Ain Shams University, Abbasiya, Cairo, 11566 Egypt; 4 Basic & Medical Sciences Department, Faculty of Dentistry, Alryada University for Science & Technology, Menoufia, Egypt; 5 Department of Operative Dentistry, Oral Functional Science and Rehabilitation Division, School of Dentistry, Asahi University, 1851 Hozumi, Mizuho, Gifu, 501-0296 Japan; 6 Department of Conservative Dentistry, Faculty of Dentistry, Universitas Indonesia, Jakarta, Indonesia; 7 Prosthetic Dentistry Department, Fixed Prosthodontic Division, Faculty of Dentistry, King Salman International University, El Tur, South Sinai, Egypt; 8 Restorative Dentistry Department, Faculty of Dentistry, Egyptian Russian University, Badr City, Cairo, Egypt

**Keywords:** Zirconia primer, 10-methacryloxydecyl dihydrogen phosphate, Silane, Self-adhesive resin cement, Weibull, Photoelectron spectroscopy, Wettability

## Abstract

**Purpose:**

To evaluate the effect of zirconia priming with MDP-Salt before MDP containing primers and self-adhesive cement on the shear bond strength.

**Materials and methods:**

Fully sintered high translucent zirconia specimens (*n* = 120) were assigned into 2 groups (*n* = 60 each): Control (No Pretreatment) and Methacryloyloxydecyl dihydrogen phosphate salt (MDP-Salt) pretreated. Each group was divided into 3 subgroups (*n* = 20) according to cementation protocol: 1) MDP + Silane primer and conventional resin cement, 2) MDP+ Bisphenyl dimethacrylate (BPDM) primer and conventional resin cement, and 3) MDP containing self-adhesive resin cement. Shear bond strength (SBS) was measured after 10,000 thermocycling. Contact angle was measured for tested groups. Surface topography was assessed using a 3D confocal laser scanning microscope (CLSM). Weibull analysis was performed for SBS and one-way ANOVA for contact angle and surface topography measurements (α = 0.05).

**Results:**

The use of MDP-Salt significantly improved the SBS (*p* < .05) for all tested subgroups. Self-adhesive cement showed an insignificant difference with MDP + Silane group for both groups (*p* > .05). MDP + BPDM showed a significantly lower characteristic strength compared to self-adhesive resin cement when both were pretreated with MDP-Salt. No difference between all tested groups in the surface topographic measurements while MDP-Salt showed the highest contact angle.

**Conclusion:**

MDP-Salt pretreatment can improve bonding performance between zirconia and MDP containing products.

## Introduction

Ceramics based on yttria-stabilized tetragonal zirconia polycrystals (Y-TZP) are gaining clinical attraction as they provide superb flexural strength, fracture toughness, excellent esthetics and high tissue biocompatibility [1, 2]. One of the challenges in dentistry is to maintain an effective and durable resin-ceramic adhesion to ensure the clinical survival of ceramic restorations.

Zirconia in particular poses as a challenge in bonding due to its polycrystalline nature. Fully sintered zirconia cannot undergo the same etching process as conventional silica-based ceramics to achieve ideal bonding to the tooth structure; hence, silane coupling agent would not be effective in achieving chemical coupling as zirconia does not have a glass phase [3, 4]. Moreover, roughening the surface using alumina blasting to obtain physical means of adhesion was not sufficient to improve the long-term adhesion with zirconia [5]. 10-methacryloxydecyl dihydrogen phosphate (MDP) primers have been introduced to achieve better adhesion and increase resin–zirconia interaction by formation of chemical bond (ionic or hydrogen) and thus improving adhesion [6]

Pure MDP on the alumina air-abraded zirconia surface is considered the gold standard for zirconia adhesion [3, 7–11]. The MDP molecule has a 10-carbons spacer ester chain with two ends; a phosphoric-acid group at one end and a vinyl group at the other end [6, 12]. The former is a metal oxides adhesion-promoter (for alumina and zirconia) and the latter facilitates polymerization with unsaturated carbon bonds in the resin matrix, while the carbon chain is hydrophobic and can withstand the hydrolytic degradation [12, 13]

To simplify the priming process to various dental substrates, adhesives or primers containing MDP mixed with other molecules in a single-bottle were developed [14–16]. However, studies have shown that the efficacy of MDP is affected when mixed with other monomers, particularly with zirconia substrate [3, 17]. Self-adhesive cements, moreover, were developed to further decrease the steps required for cementation and showed promising results with zirconia [11, 18]

A new MDP-salt was introduced as a cleaner and adhesion promotor for zirconia [5, 19]. The priming effect of MDP-salt was introduced in early 2022 [5], however, the effect of MDP-Salt combined with different MDP primers and MDP containing self-adhesive cement on the bonding performance to zirconia surface has never been reported. Therefore, the purpose of this study was to evaluate MDP-salt pretreatment before primer application and self-adhesive resin cement. The null hypotheses tested in this study were: 1. bonding to zirconia will not be influenced by MDP-Salt pretreatment, 2. different MDP-based primers and cement will show no difference in the bonding performance to zirconia.

## Materials and methods

High translucent yttria-stabilized tetragonal zirconia polycrystals (5Y-PSZ; Liaoning Upcera Co., Ltd. Liaoning, China) were cut into square specimen (10 × 10 × 3 mm) using a low-speed diamond wafering blade mounted on precision saw machine (Isomet 4000, Buehler, Lake Bluff, IL, USA). Another rod from the same material was milled with 2 mm radius and then cut into 3 mm thick discs to be cemented to a counterpart square specimen. The full composition of used materials is listed in Table 1.

**Table 1:** Materials used in the current study

Material	Manufacturer	Composition [Batch]
TT ML MT (B2 D98–16) (Sintered)	Liaoning Upcera Co., Ltd. Liaoning, China	86.3–94.2 wt% ZrO_2_ + HfO_2_, 5.8–9.7 wt% Y_2_O_3_, < 0.5 wt% Al_2_O_3,_ < 2.0 wt% Er_2_O_3_, < 0.5 wt% other oxides [L2190905167–48]
Alumina (Al_2_O_3_)	Kulzer,Japan	Al_2_O_3_ 50 μm
MDP-Salt	Kuraray Noritake Dental, Japan	10-MDP, triethanolamine, Water, Colorant (pH ~ 4–5) [370027]
Visalys restoration primer [MDP + Silane]	Kettenbach GmbH & Co. KG, Eschenburg, Germany	10-MDP, Silane coupling agent, Ethanol [210151]
Z-prime [MDP + BPDM]	Bisco Inc., IL, USA	10-MDP, BPDM (Bis-GMA, HEMA) Ethanol [2100007462]
Visalys CemCore	Kettenbach GmbH & Co. KG, Eschenburg, Germany	UDMA, other Dimethacrylate (aliphatic Trimethacrylate / aliphatic Dimethacrylate), Ytterbium fluoride and silica Polymorph, Benzoyl peroxide [210331006]
TheraCem	Bisco Inc., IL, USA	Base: Calcium base filler, glass filler, Bis-GMA, dimethacrylates, 2-hydroxyethyl methacrylate, ytterbium fluoride, initiator, amorphous silica Catalyst: Glass filler, 10-MDP, amorphous silica [2100008614]

10-MDP; 10-methacryloyloxydecyl dihydrogen phosphate. HEMA; 2-Hydroxyethyl methacrylate monomethyl ether. Bis-GMA; Bisphenol A di (2-hydroxy propoxy) dimethacrylate. UDMA; urethane dimethacrylate

### Specimen preparation

For shear bond strength test: at total of 120 squares 5Y-PSZ specimens and 120-disc specimens were sintered at 1450 C^0^ according to the manufacturer’s instructions in a high-temperature furnace (Lindberg/Blue M, Asheville, NC, USA). All the specimens were polished with 600-grit silicon carbide (SiC) paper then blasted with 50-μm Al_2_O_3_ particles (Kulzer, GmbH, Germany) at 25 psi for 20 seconds at a 10 mm distance and 90^0^ angle using a sandblasting device (Microetcher IIA, Danville Materials, SanRamon, CA, USA).

**Fig. 1:**
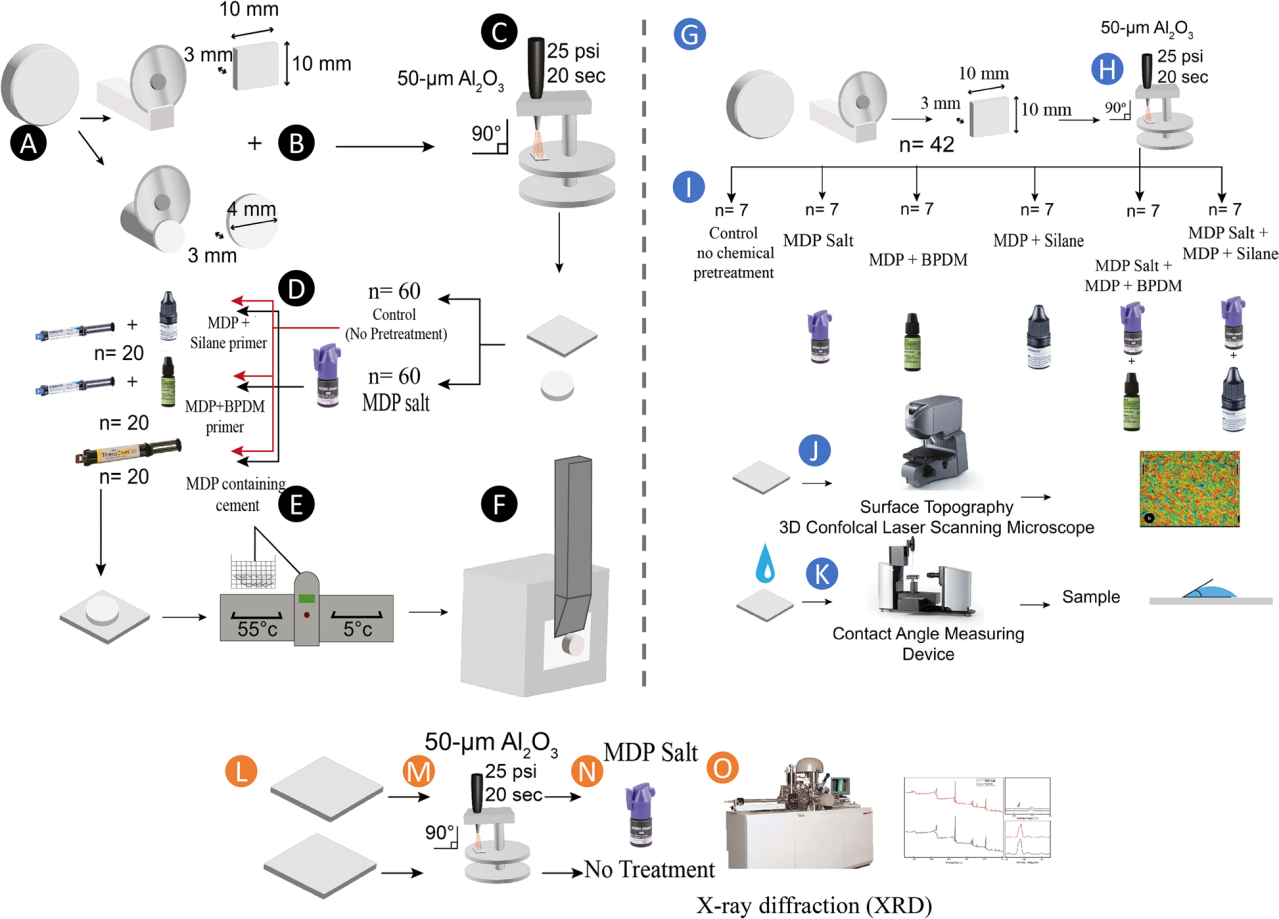
Diagrammatic illustration for the experimental steps. **A**-**F** Illustrates the steps for shear bond strength testing. **A** 5Y-PSZ zirconia substrate. **B** Square and disc-shaped specimens were prepared and sintered **C** All specimen were sand-blasted with 50 μm Al_2_O_3_. **D** Specimens divided into 2 groups according to pretreatment (Control and MDP-Salt pretreated groups) and furtherly divided according to the cementation protocol [grouping explained in section 2.1]. **E** Cemented specimens were thermocycled for 10,000 cycles. **F** Shear bond strength testing using universal testing machine. **G**-**K** Illustrates the steps for surface roughness and contact angle measurements. **G**-**H** Square-shaped 5Y-PSZ zirconia specimens prepared and sand-blasted. **I** specimens were divided into 6 groups according to pretreatment and primer application [grouping explained in section 2.3]. **J** Specimens were tested for surface roughness using 3D confocal laser scanning microscope. **K** Specimens were tested for contact angle measurement. **L**-**O** Illustrates the steps for x-ray diffraction measurements (XRD). **L** Square-shaped specimens prepared. **M** Specimens were sand-blasted. **N** Specimens were divided into 2 groups (control: no pretreatment and MDP-Salt groups). **O** Specimens were tested using XRD

All specimens were cleaned ultrasonically for 2 minutes in distilled water followed by drying with oil-free air. Each large square-shaped specimen was bonded to the smaller round-shaped specimen. Specimens were divided into 2 groups (*n* = 60) according to the pretreatment protocol into; Control (No Pretreatment) and MDP-Salt Salt (application: 1 min rubbing the specimen surface with MDP-Salt [Katana Cleaner, Kuraray Noritake Dental, Japan] then washed with water spray for 1 min). Each group was divided into 3 subgroups based on the cementation protocol as follow; 1) MDP + Silane: the specimens were primed with Visalys restorative primer (Kettenbach GmbH & Co. KG, Eschenburg, Germany) for 60 seconds then a gentle jet of air was applied, then conventional resin cement (Visalys CemCore, Kettenbach GmbH & Co. KG, Eschenburg, Germany) was used to cement the disc-shaped zirconia specimen’s counterpart. Light curing was done using LED (1500 mW /cm^2^, 18 CuringPen, Sifary medical technologies, Jiangsu Province, China) for 40 seconds. 2) MDP + BPDM: the specimens were primed with 2 coats of Z-Prime Plus (BISCO Inc. 1100 W Irving Park Rd.Schaumburg, IL USA) for 5 seconds then a gentle jet of air was applied, then cementation with conventional resin cement and curing similar to MDP + Silane subgroup. 3) MDP containing cement: self-adhesive resin cement (TheraCem, BISCO Inc. 1100 W Irving Park Rd.Sshaumburg, IL USA) was used for cementation of the disc-shaped zirconia specimen counterpart without primer and Light cured using LED (1500 mW /cm^2^, 18 CuringPen, Sifary medical technologies, Jiangsu Province, China) for 40 seconds. Specimens’ preparation and grouping for shear bond strength testing explained in Fig. 1(A-F).

### Shear bond strength (SBS) testing

All specimens were thermocycled for 10,000 cycles before SBS testing. SBS was tested using a universal testing machine (Instron, model 3345, England) with a crosshead speed of 1 mm/min. The debonded interface of the specimens was examined after fracture under a stereo microscope (20×; Olympus, Tokyo, Japan) and mode of failure were categorized as; “C” for cohesive failure at the resin cement, “A” for adhesive failure at the ceramic-resin interface, and “M” for mixed failure (more than 30 to70% of C or A).

### Surface topography examination

5Y-PSZ square specimens (*n* = 42) were prepared as described in the specimen preparation section to be used in surface topography examination and contact angle measurements. All specimens were alumina blasted. They were assigned into 6 groups (*n* = 7) according to surface treatment as follow; 1) Control (alumina blasted- no chemical pretreatment); 2) MDP Salt, 3) MDP + BPDM, 4) MDP + Silane, 5) MDP Salt+ MDP + BPDM, and 6) MDP Salt+ MDP+ Silane. The surface topographic measurements of the tested groups were measured using a 3D confocal laser scanning microscope (CLSM, Keyence VK-X100, Keyence, Japan) with a 50X lens (scanning area 205 × 273.3 μm). The scanned surface plots were processed with a MultiFile Analyzer (V.1.3.1.120, Keyence). Specimens’ preparation and grouping for surface topography and contact angle testing explained in Fig. 1(G-K).

### Contact angle (CA) measurement

The same samples and grouping used for surface topography examination were used for contact angle measurements. The surface wettability of deionized water was examined using the sessile drop method and the contact angle was measured with a contact angle measuring device (DSA25B, Krüss GmbH, Germany). Three drops were measured, and the average was considered the mean of each specimen which was reported for statistical analysis.

### Surface elemental analysis

To evaluate the effect of MDP Salt on the zirconia surface, X-ray photoelectron spectroscopy (XPS, JPC-9010MC, JEOL, Tokyo, Japan) was used for MDP Salt treated specimen and untreated one (control). The Mg Kα X-ray source runs under the following conditions:

- Operating pressure = 10^−7^ Pa.
- Emission current = 10 mA.
- Accelerating voltage = 10 kV a.
- Pass energy setting = 100 eV.

The chemical states were identified based on peak positions and separations of the high-resolution scans for zirconium, oxygen, carbon, aluminum, silicon, and phosphorus peaks from the surface of the specimens. Specimens’ preparation and grouping for surface elemental analysis explained in Fig. 1(L-O).

### Statistical analysis

The minimum sample size for SBS was determined based on a pilot study and a previous published work [18] to be 66 specimen (*n* = 11, each group) as a large effect size resulted (f = 0.592) when the α = 0.05 to detect a power of 95%. The sample size was increased in each group to 20 specimens for the validity of the reliability analysis conducted in the current study. SBS data were statistically analyzed using Weibull analysis (R4.1, R: A language and environment for statistical computing. R Foundation for Statistical Computing, Vienna, Austria). Maximum Likelihood estimation was used for Weibull parameters calculation. The different groups were compared at the characteristic strength (63.2% probability of failure). Following confirmation of the normal distribution with the Shapiro-Wilk test, one-way analysis of variance (ANOVA) was used to compare mean contact angle and surface parameters values, followed by multiple comparisons with Bonferroni correction (α = 0.05).

## Results

### Shear bond strength (SBS)

**Fig. 2:**
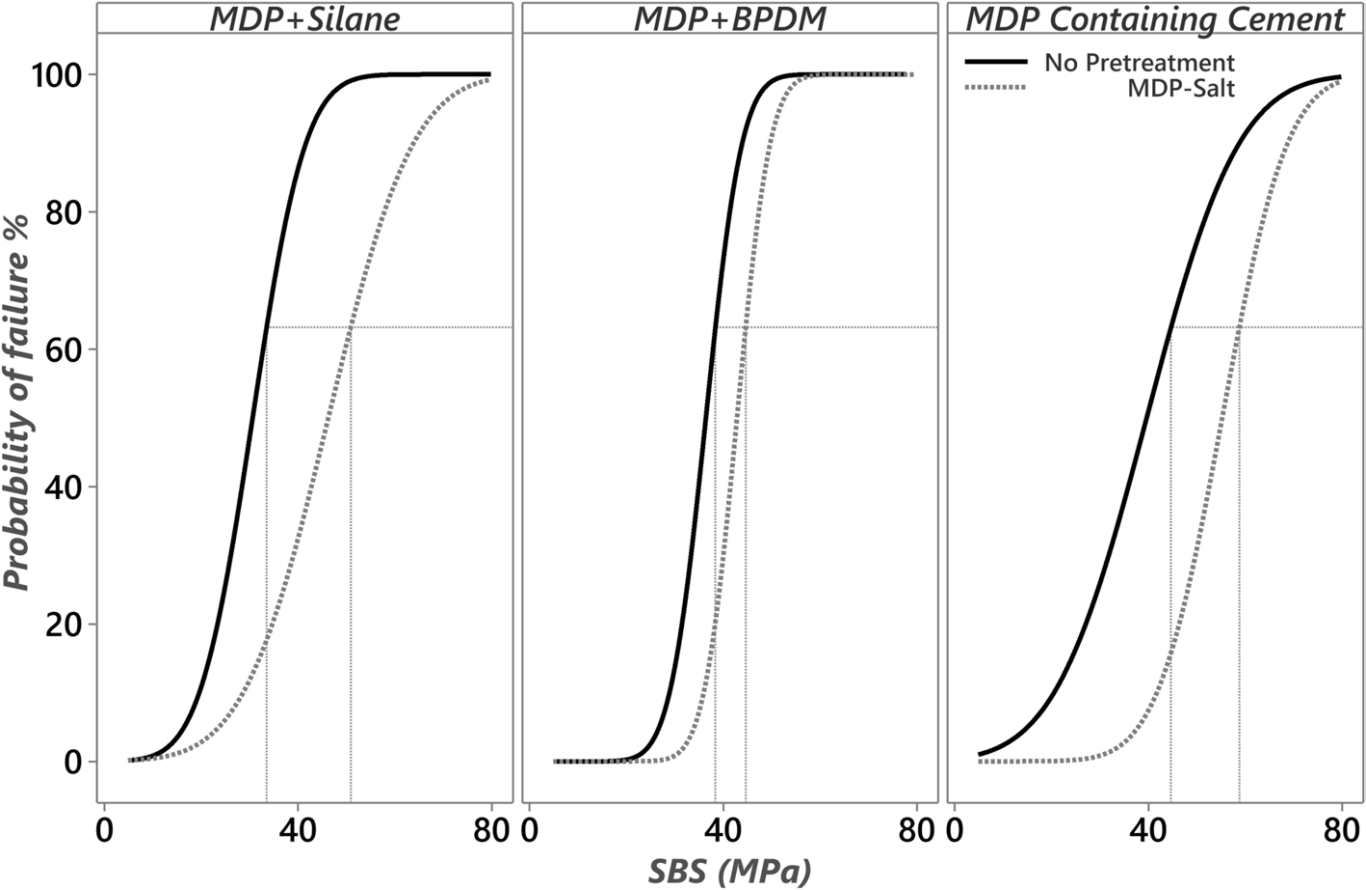
The Weibull survival graphs of the shear bond strength (MPa) of the tested groups. A horizontal dashed line at 63.2% probability of failure helps to compare the characteristic strengths of the tested groups with the corresponding vertical dashed line. MDP-Salt pretreatment showed higher characteristic strength compared to no-pretreatment for all groups

The results of the Weibull analysis are presented in Table 2 and Fig. 2. MDP-Salt pretreatment resulted in significantly higher characteristic strength compared to no-pretreatment group for all cementation protocols at *p* < 0.05. For no-pretreatment group, insignificant difference between cementation protocols were reported (*p* > 0.05). For MDP-Salt pretreated groups, self-adhesive cement showed a higher significant characteristic strength compared to MDP + BPDM.

**Table 2:** The results of Weibull analysis and failure modes distribution

Promotor	Primer	Cement	α [95% CI]	β [95% CI]	P10 [95% CI]	ptf	FM [A/C/M]
No Pretreatment	MDP + BPDM	Conventional	38.8^a^[35.7 to 42.1]	8.2[5.3 to 18.1]	29.5[22.3 to 34.1]	0/20	[90/0/10]
No Pretreatment	MDP + Silane	Conventional	33.7 ^a^[28.3 to 40.2]	4.0[2.7 to 7.6]	19.1[11.2 to 25.6]	0/20	[100/0/0]
No Pretreatment	–	Self-adhesive	43.7 ^ab^[35.3 to 53.7]	3.2[2.0 to 7.7]	21.4[9.7 to 31.0]	0/20	[80/0/20]
MDP-Salt	MDP + BPDM	Conventional	45.0^b^[42.4 to 47.9]	11.3[7.2 to 26.0]	36.9[29.9 to 41.0]	0/20	[100/0/0]
MDP-Salt	MDP + Silane	Conventional	51.0^bc^[44.0 to 59.2]	4.4[2.9 to 9.3]	30.6[18.5 to 39.6]	0/20	[100/0/0]
MDP-Salt	–	Self-adhesive	59.5^c^[54.0 to 65.9]	6.4[4.3 to 12.6]	41.9[30.3 to 49.9]	0/20	[90/0/10]

Different superscript letters within the α column are statistically significant differences based on a 95% confidence interval (CI). α: characteristic strength or scale of a Weibull parameter. β: the shape, slope, modulus of a Weibull parameter. P10: estimation at 10% probability of failure. FM: failure modes percentage; (A) adhesive failure at ceramic-resin interface, (C) cohesive failure at the resin cement, and (M) mixed failure

For all tested groups, no pretest failure resulted, and failure mode showed 80–100% adhesive failure.

### Surface topography

**Fig. 3:**
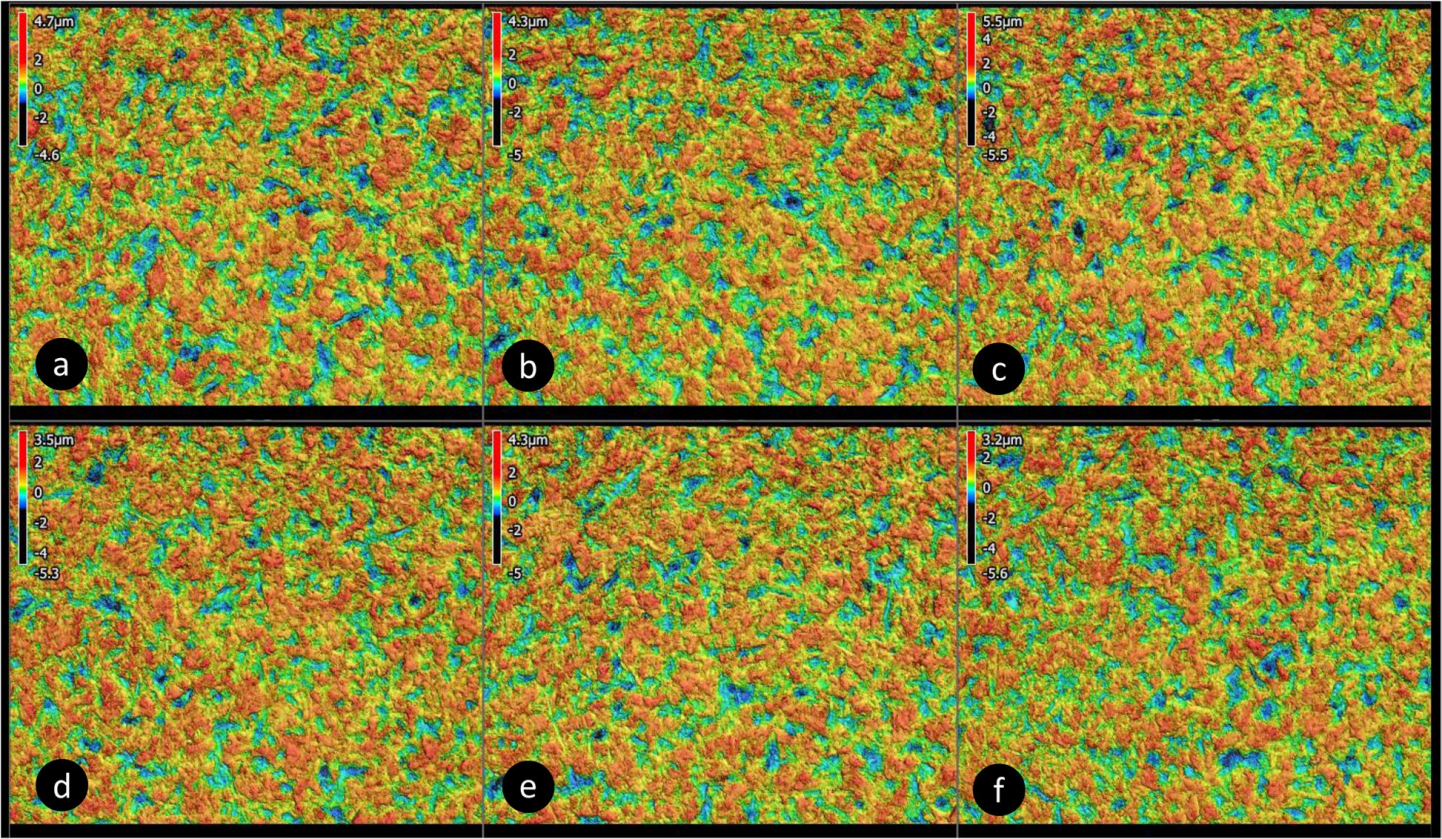
confocal microscope Laser scanning 3D (3D-CLSM) topography images for different groups (50x magnification). **A** control, **B **MDP-Salt, **C** MDP + BPDM, **D** MDP + Silane, **E** MDP-Salt+MDP + BPDM, and **F** MDP-Salt+MDP + Silane

**Fig. 4:**
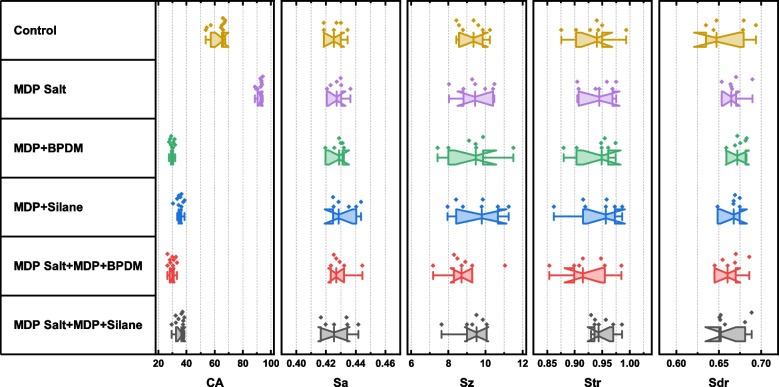
(CA) Contact angle measurement of different tested groups. MDP-Salt resulted in higher CA. (Sa) Arithmetical mean height, (Sz) Sum of the largest peak heigh, (Str) Texture aspect ratio, and (Sdr) Developed interfacial area ratio. Insignificant difference between groups for all topographic parameters

A representative CLSM image is presented in Fig. 3 and results of surface roughness parameters are presented in Table 2 and Fig. 4. For all tested groups there was an insignificant difference in arithmetical mean height (Sa), maximum height (Sz), texture aspect ratio (Str) and developed interfacial area ration (Sdr) parameters (*p* > 0.05).

### Contact angle (CA)

The contact angle results are displayed in Table 3 and Fig. 4. Zirconia substrates pretreated with MDP-Salt showed significant increase in the CA compared to control. The application of the MDP-Salt for 1 min followed by a water stream for cleansing, leaves the zirconia surface hydrophobic. MDP + BPDM and MDP-Salt+MDP + BPDM showed the lowest CA with insignificant difference between each other. The application of MDP + Silane showed an increase in the contact angle measurement compared to the MDP + BPDM group.

**Table 3:** Contact angle (CA) measurements and surface roughness parameters

	CA	Sa	Sz	Str	Sdr
Control	62.323^b^ ± 5.587	0.417 ± 0.025	9.265 ± 0.687	0.933 ± 0.037	0.624 ± 0.105
MDP Salt	91.953^a^ ± 2.001	0.417 ± 0.031	9.494 ± 0.884	0.942 ± 0.028	0.629 ± 0.104
MDP + BPDM	29.772^d^ ± 1.436	0.416 ± 0.031	9.268 ± 1.323	0.938 ± 0.034	0.632 ± 0.108
MDP + Silane	35.253^c^ ± 2.433	0.423 ± 0.027	9.534 ± 1.199	0.939 ± 0.043	0.627 ± 0.106
MDP Salt+MDP + BPDM	29.592^d^ ± 2.441	0.42 ± 0.028	8.849 ± 1.173	0.923 ± 0.043	0.624 ± 0.108
MDP Salt+MDP + Silane	35.04^c^ ± 3.1	0.419 ± 0.028	9.696 ± 1.475	0.951 ± 0.021	0.625 ± 0.103
*p*-value	< 0.001	0.934	0.714	0.805	0.923

Different letters with each CA column indicate significant difference

### XPS

**Fig. 5:**
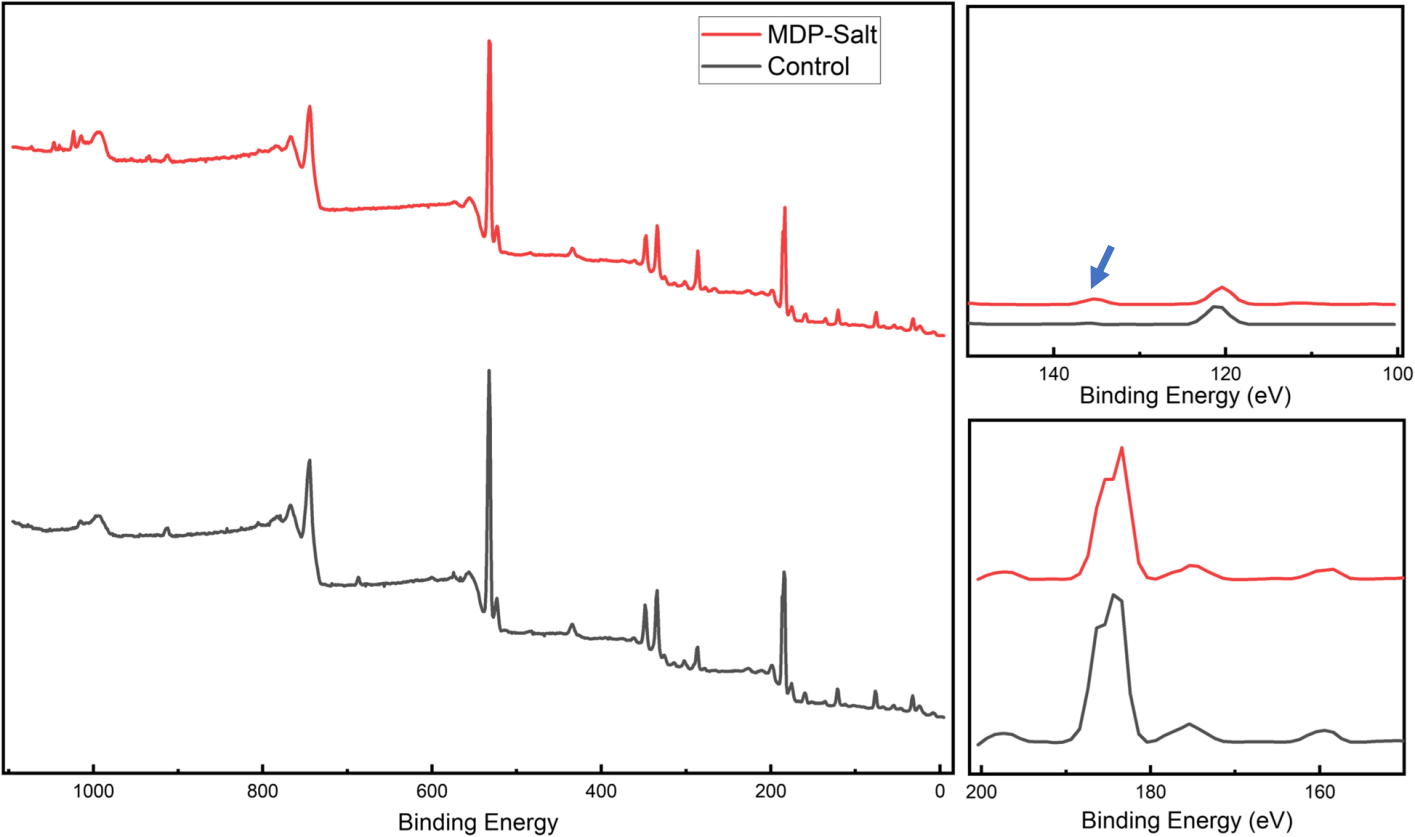
Wide scan XPS spectra for alumina air-blasted zirconia ceramics (Black) and MDP-Salt primed alumina air-blasted zirconia ceramics (red). The zirconium (Zr3d, Zr3p) and aluminum (Al2p, Al2s) peaks were detected in both groups. The phosphorous (P2p-blue arrow) peak was visible for MDP-Salt treated substrate

Wide-scan spectra from XPS analysis are presented in Fig. 5. Alumina air-blasted zirconia whether pretreated with MDP-Salt or not showed similar spectra except that phosphorus peak was detected on MDP-Salt. Both spectra showed Zr and Al peaks.

## Discussion

In the current investigation, we examined the effect of MDP-Salt application prior to MDP primers application and MDP containing self-adhesive cement on the bonding performance to zirconia substrate. Based on the current results, the first null hypothesis was rejected as MDP-Salt improved the bonding performance with all MDP containing primers/cement.

The tested zirconia primers were MDP-based, with silane or with carboxylic based monomer (BPDM). Both showed similar bonding performance to each other which may be attributed to the presence of MDP as the functional molecule. Additionally, both primers benefited from the pretreatment with MDP-Salt due to increasing the amount of attached MDP molecules to zirconia surface [5]. 1 minute pretreatment with MDP-Salt followed by washing with water stream can clean and prime zirconia surface [5, 19]. A phosphorus peak on XPS was detected for MDP-Salt pretreated substrate (Fig. 5) confirming the priming action of MDP-Salt. Triethanolamine in MDP-Salt is responsible for cleaning the surface by attaching to the surface contaminants [19] and washing them away which improves the interaction between clean zirconia/aluminum oxide and phosphate of MDP with the presence of (−OH) from the three ethanol molecules [5]. After primer applications, MDP from the primer furtherly attaches to the surface of the vacant (−OH) in both zirconia surface and triethanolamine making a more reliable priming effect for the zirconia substrate. The latter resulted in a significant increase in CA after MDP-Salt application, confirming the increased MDP attachment to the zirconia surface. Surface topography was insignificantly different between all tested groups which indicates that MDP-Salt and other primers did not alter the mechanical roughness parameters of alumina air-blasted zirconia surface.

MDP containing self-adhesive resin cement outperformed zirconia primed with MDP+ BPDM when MDP-Salt was used. It may be attributed to the MDP hydrolysis during storage which is promoted by methacrylate products in MDP + BPDM primer [17, 20]. While for self-adhesive cement used in the current study, MDP and methacrylate products are stored in separate compartments and freshly mixed immediately before cementation, which prevents the MDP hydrolysis. Thus, the second null hypothesis was partially rejected.

**Fig. 6:**
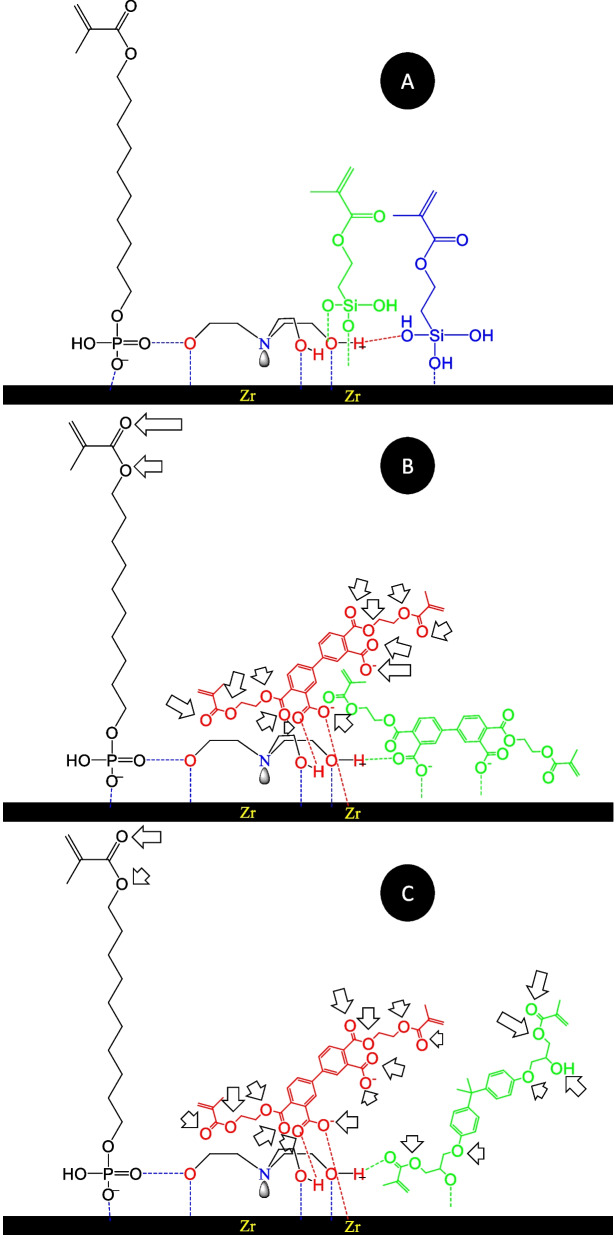
**A** The Proposed structure for 10-MDP (black) and silane (green and blue) with MDP-Slat on zirconium surface. **B** The proposed structure for 10-MDP (black) and BPDM (red and green) with MDP-Salt on zirconium surface **C** the proposed structure for 10-MDP (black, BPDM (red), and BIS-GMA (green) with MDP-Salt on zirconium surface. The black arrows show the polar site that can make H-bond with water (decrease CA (Table 3 and Fig. 3))

The idea of using MDP primer prior to cementation with MDP containing self-adhesive cement seems similar to MDP-Salt application, however, previously, MDP + BPDM primer application prior to MDP contained resin cements didn’t showed an improvement in the bonding performance to zirconia [21]. MDP containing self-adhesive resin cement benefits from the MDP-Salt priming action compared to primer application prior cementation with MDP containing self-adhesive resin cement. The difference between MDP-Salt and other primers is attributed to application procedure, as for MDP-Salt, the excess material is washed away from the zirconia surface with water stream leaving only the reacted layer of MDP on the surface. On the other hand, MDP + Silane and MDP + BPDM primers application leaves an excess unreacted layer of primer monomers [3, 22, 23] which results in similar results for CA when applied directly or preceded with MDP-Salt pretreatment. Both MDP + BPDM and MDP + Silane showed a decrease in the CA, compared to control or MDP-Salt, due to the increased polar sites that make hydrogen bond with water (Fig. 6). The double bond sites are responsible for the polymerization with other resin monomers and create the bond between primers and resin cement. The CA measurement was done to explain the functional groups and their interaction with zirconia surface rather than explaining the bond strength variations in the tested groups [5, 19].

The current research is the first to investigate the use of MDP-Salts as an adhesion promotor prior to MDP-containing primers/cements which limits the current work to explanation of the theoretical assumption rather than comparison with earlier published data. Further research still needed to investigate the effect of MDP-salt with longer storage time and hydrothermal aging. Additionally, clinical research still needs to confirm our laboratory findings.

## Conclusion

Within the limitation of this study, we can conclude that:

1. MDP-salt can be applied as an adhesion promoter to zirconia prior to primer applications to improve zirconia bonding performance.
2. One-step cementation with MDP containing self-adhesive resin cement can be an effective alternative to 2-step cementation which requires a separate priming step.
3. MDP containing self-adhesive resin cement can also benefit from MDP-Salt pretreatment.

## Data Availability

Dataset used and analyzed data can be available form corresponding author on reasonable request.
